# Can the Hole–Board Test Predict a Rat’s Exploratory Behavior in a Free-Exploration Test?

**DOI:** 10.3390/ani11041068

**Published:** 2021-04-09

**Authors:** Wojciech Pisula, Klaudia Modlinska, Katarzyna Goncikowska, Anna Chrzanowska

**Affiliations:** Institute of Psychology, Polish Academy of Sciences, Jaracza 1, 00-378 Warsaw, Poland; kmodlinska@psych.pan.pl (K.M.); katarzyna.goncikowska@gmail.com (K.G.); achrzanowska@psych.pan.pl (A.C.)

**Keywords:** hole–board, free-exploration test, exploratory behavior, rat

## Abstract

**Simple Summary:**

Since the introduction of the hole–board test, its validity and applicability have been repeatedly re-examined. The hole–board protocol remains one of the standard procedures applied in psychopharmacology and behavioral studies. Some authors advocate the use of the hole–board procedure in studies on various aspects of behavior regulation, such as exploration and anxiety, habituation to a novel environment, spatial learning and memory (working and reference memory), spatial pattern learning, and food search strategies. In this study, we focused on rats’ activity in the hole–board test that we considered to be a type of exploratory activity. Based on our results and our previous studies of rats’ exploratory behavior in the free-exploration box, we suggest that the hole–board apparatus might not be the best tool for measuring exploratory behavior in laboratory rodents.

**Abstract:**

This study focuses on the rat activity in a hole–board setting that we considered a type of exploratory behavior. The general hypothesis is based on the claim that a motivational mechanism is central to both the response to novelty in a highly familiarized environment and the activity in the hole–board apparatus. Our sample consisted of 80 experimentally naive Lister Hooded rats. All rats were tested in the hole–board apparatus. Twenty individuals with the highest hole-board scores and twenty subjects with the lowest hole–board scores subsequently underwent an established free-exploration test. In our study, the scores obtained in the hole–board test had little predictive value for the rats’ activity in the free-exploration test. Based on our previous experience in studying exploratory behavior in the free-exploration test and the data presented in this paper, we suggest that the hole–board test is not an appropriate tool for measuring exploratory behavior in laboratory rodents.

## 1. Introduction

The hole–board test is a behavioral test that has been used to assess different aspects of cognitive abilities and emotions in small mammals. It originated from an open field test, which was designed to evaluate exploratory behavior and anxiety [1]. The hole–board apparatus has small cylindrical holes at the bottom of the experimental arena that allows experimenters to conduct more complex behavioral observations. A head-dipping in the holes is considered a main feature of the behavior in this experimental arena. However, since the introduction of the hole–board test [2], its validity and applicability have been repeatedly re-examined. Head-dipping in the hole was considered by File and Wardill [3] to be a valid measure of exploratory activity. The discussion continued, and R. Hughes [4] (p. 449) stated: “There is also the possibility that head-dipping could involve attempts to find an escape route rather than reflect a genuine interest in objects underneath the holes”. This view was substantiated by an experimental study on Lister-Hooded rats [5] (p. 442) in which the authors concluded: “Rather than being a measure of neophilia, these results support the hypothesis that head-dipping represents an escape response, which declines as the subject becomes less fearful”. Despite this controversy, the hole–board protocol remains one of the standard procedures applied in psychopharmacology and behavioral studies. Some authors [6] advocate the use of the hole–board procedure in studies on various aspects of behavior regulation (such as exploration and anxiety, habituation to a novel environment, spatial learning and memory (working and reference memory), spatial pattern learning, and food search strategies) [7,8]. More recently, the hole–board procedure has also been used to assess behavioral characteristics that are supposed to reflect an animal model of Autism spectrum disorder (ASD) [9].

Neophillia (the tendency to approach unfamiliar objects or environments—[10,11]) in animal behavior has long been a subject of scientific discussion. We share the view expressed in earlier papers that any behavior in a novel environment would be regulated by both neophilia and neophobia [5,6,12,13]. Therefore, rather than being at the two extremes of a continuum, neophilia and neophobia should be thought of as two independent mechanisms that can come into play simultaneously. Over the past three decades, research on curiosity and exploration in animals has established some basic methodological guidelines for further studies. The experimental stimulus of novelty should be controlled by ensuring a sufficiently long habituation period. Moreover, the test environment should provide the subjects with a comfortable low-stress setting, which does not trigger neophobic or defense responses. A validated protocol of this methodological approach has already been established [14], and a detailed description of the free-exploration test was published [15], enabling readers to fully replicate all of the procedural details. Since the ecological validity of the protocol for neophillia and the exploratory responses has been clearly demonstrated, it suggests that the free-exploration test may serve as a tool for evaluating other methods of behavioral assessments in terms of their validity for measuring exploratory behavior. Since the hole–board procedure is often used as a tool for measuring exploratory behavior in rodents, our study sought to validate the two protocols in question, namely the hole–board protocol and the free-exploration test (low-stress), as previously described [15].

To date, there are many variants of the hole–board devices. We chose the most standard and widely used apparatus. While the hole–board procedure is used for numerous purposes (such as anxiety or general activity assessment or behavioral profiling), we have focused on the exploratory aspect of the behavior in the rats’ activity on the hole–board. The general hypothesis was based on the claim that there is a motivational mechanism central to both the response to novelty in a highly familiarized environment and the activity in the hole–board apparatus. If it is true, there should be a strong positive correlation between both kinds of activity, namely the response to novelty in the free-exploration box and the activity in the hole–board apparatus.

## 2. Materials and Methods

### 2.1. Animals

The sample consisted of 80 experimentally naive Lister Hooded rats. The rats were bred and kept in the vivarium of the Institute of Psychology, Polish Academy of Sciences, Warsaw, Poland. The rats were approximately 90 days old and weighed approximately 350 g at the start of the experiment.

The rats were housed in groups of 3–4 in Tecniplast Eurostandard Type IV cages (610 mm × 435 mm × 215 mm) with dust-free softwood granules (Tierwohl Super, Rosenberg, Germany) as bedding and with ad libitum access to water and standard laboratory fodder (Labofeed H, WP Morawski, Kcynia, Poland). The day/night cycle was set at 12/12 h (with the lights on at 8 a.m.) and the temperature was maintained at a constant 21–23 °C. The cages and pens were cleaned once a week, on the same day and at a fixed time, in the late afternoon (5 p.m.). However, in order to ensure that the experimental procedure was not disturbed, the cages in which the test animals were kept were cleaned just before the onset of the experiment and again after the experiment was finished. All of the rats kept in our laboratory were housed, bred and taken care of in accordance with the Regulation of the Polish Minister for Agriculture and Rural Development of 14 December 2016 on laboratory animal care. The experimental procedures were approved by the First Local Ethics Committee on Animal Experimentation in Warsaw, Poland (No. 1116/2020).

The sample size was estimated using a commonly used formula for calculating sample size for repeated measures [16]:

N = 1 + C(s/d)^2^

where:

s—standard deviation of the population means

d—size of difference in means (the effect)

C—constant dependent on the value of α (significant level) and 1-ß (power)

For the purpose of our study, we employed the following parameters: α = 0.05; β = 0.20 (C = 10.51).

Group size calculations were carried out on the basis of a previous study [14] in which the average time spent on exploring changed objects was M = 96.51, with standard deviation s = 36.63, and on the assumption that the detectable difference between the variables should be d = 41.

Therefore, the total sample size for the free-exploration test was estimated at 10.

### 2.2. Procedure

The experiment was conducted in two phases. In Phase I, a hole–board test was carried out. Then, the selected animals were subjected to the exploration test in Phase II.

#### 2.2.1. Phase I—The Hole–board Test

The aim of the test was to measure the rats’ activity (i.e., head-dipping behavior) in the experimental area and select individuals with the lowest and the highest levels of analyzed behavior. The scores were assigned based on the number of instances of head-dipping in the holes during a 5-min session. Twenty subjects (10 males and 10 females) with the highest scores and twenty individuals (10 males and 10 females) with the lowest scores on the hole–board test were admitted to Phase II.

**Figure 1:**
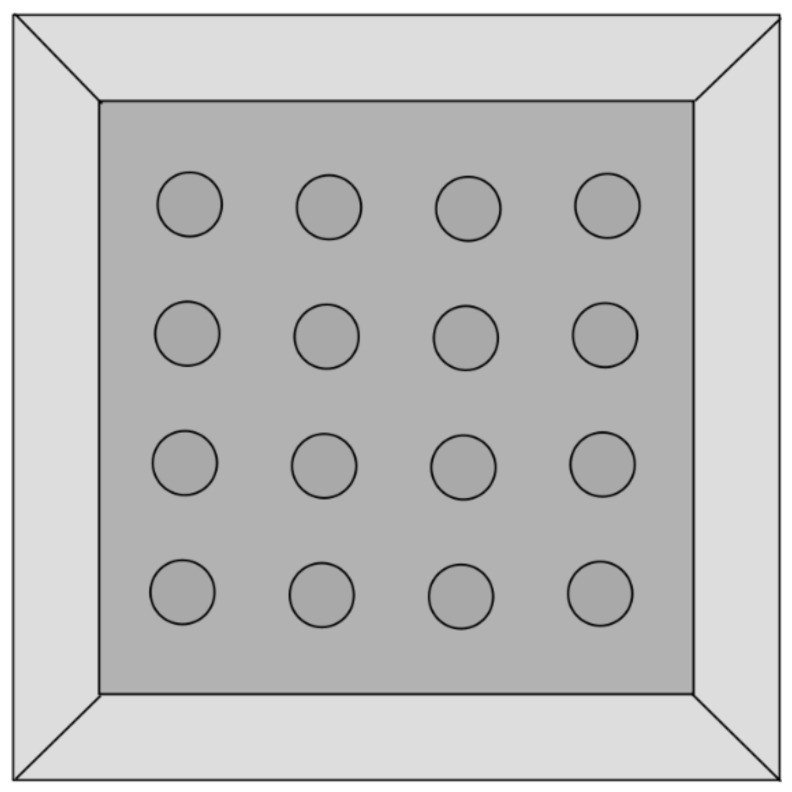
Hole–board apparatus (view from above).

We used a square hole–board arena measuring 600 × 600 × 450 mm (Hole–board for rats, manufactured by MazeEngineers, Skokie, IL, USA) (Figure 1). The transparent Plexiglas walls were covered to prevent the animals from being distracted by visual stimuli. There were 16 round-shaped holes in the bottom of the arena, each 50 mm in diameter, distributed evenly at equal distances from each other. The experimental arena was illuminated by fluorescent ceiling lamps, set at approximately 75–100 lx. The video camera used for recording the rats’ behavior was placed 1.2 m above the measurement apparatus.

After being taken out of the housing cage, each rat was immediately placed in a transporter (a small cylindrical cage 60 mm in diameter with doors 120 mm high and 100 mm wide) and moved to another room where the experiment took place. The transporter with the animal was placed in the corner of the experimental arena in such a way that the rats could leave the transporter through the exit into the interior of the arena. After the transporter exit was opened, each animal was left in the arena for five minutes. Each rat was free to explore the experimental arena and leave and enter the transporter without impediment. After completing the session, the animal was removed from the experimental arena and returned to its home cage. The hole–board test was carried out once. The testing arena was cleaned after each rat using Virkon S (Bayer, Leverkusen, Germany).

#### 2.2.2. Phase II—The Free-Exploration Test

One day after the hole–board test, the selected (high-activity and low-activity) rats participated in the free-exploration test. The high-activity group included 20 individuals (10 males and 10 females) that had the highest scores in the hole–board test whereas the low-activity group included another 20 individuals (10 males and 10 females) that had the lowest scores in the test.

The aim of the free-exploration test was to measure the differences between the process of exploring the new environment, the rate of habituation to it, and the response to the introduction of harmless novelty into the familiar context. The apparatus and the measurement methods were similar to those used in our previous studies (e.g., [14,15,17,18,19]).

**Figure 2:**
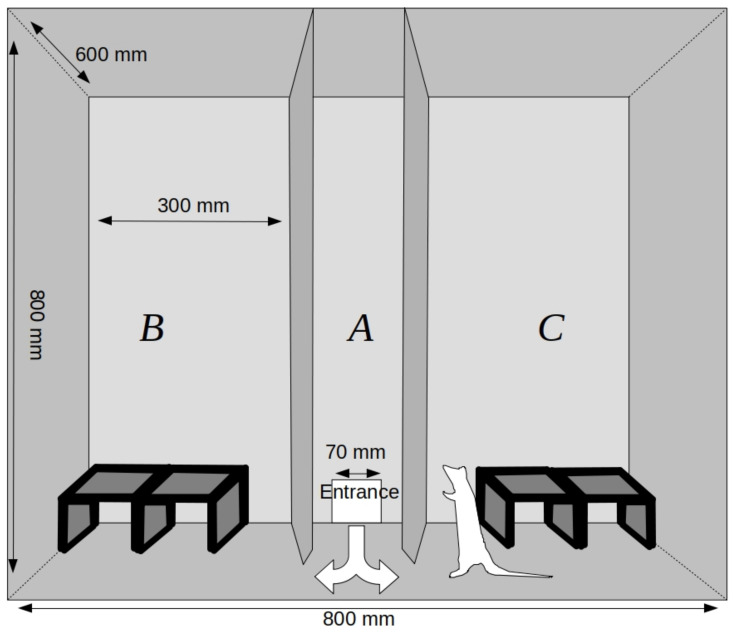
The experimental chamber of the free-exploration test—frontal view through the transparent front wall.

The experimental chamber was a box with dimensions of 800 mm × 600 mm × 800 mm. The space inside the chamber was divided into three zones (A–C), separated by two walls diametrically perpendicular to its longer side (Figure 2). The front part of the chamber was a transparent wall that could be elevated in order to gain full access to the testing arena. The wooden partition walls between the zones had triangular passages (120 mm × 140 mm) at the bottom, which allowed animals to move freely between parts of the chamber. The entire chamber was coated with a layer of removable varnish. Wooden tunnels (200 mm × 120 mm × 80 mm) covered with washable paint were positioned in zones B and C. Unlike the most commonly used two-dimensional experimental settings, these tunnels provided a complex three-dimensional environment. The middle zone (A) was empty. There was a hole in the back wall of the chamber that functioned as an entrance for animals moving from the animal transporter to the chamber. The transporter functioned as a starter box (60 mm in diameter with doors 120 mm high and 100 mm wide).

**Figure 3:**
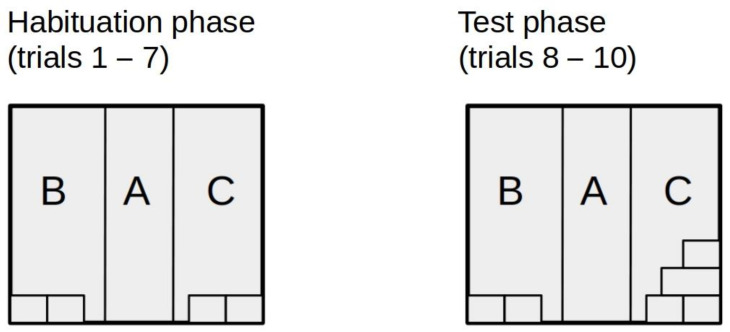
The arrangement of objects in the experimental chamber in each experimental setting. Habituation phase—two tunnels placed next to each other in sections B and C; Test phase—two tunnels placed next to each other in section B and two tunnels placed next to each other and an additional two tunnels placed at the top in section C.

At the beginning of each trial, the transporter with the animal inside was placed at the entrance to Zone A. Then, the rat was left unhindered in the starting box for 15 s, after which the entrance door was opened. The animal was allowed to stay in the starting box or leave it to examine the chamber. The first seven trials were habituation trials, during which the apparatus was adjusted in exactly the same way (Figure 3). The implementation of the novelty took place on trial 8. The novelty took the form of adding additional tunnels, as it is shown in the right panel of Figure 3. Three consecutive tests trials were carried out with the chamber in this new configuration.

Each trial lasted 7 min and was carried out for each animal once a day. Each experimental session was followed by a cleaning of the experimental arena, the tunnels, and the transporter using Virkon S (Bayer) in order to eliminate odors left by the previous animal.

A video camera was placed at a distance of approximately 1.5 m from the transparent front wall of the experimental chamber. The camera was set up in night-time shooting mode to ensure the possibility of filming in the dark.

To prevent the effects of lateralization or visual/auditory cues, novel objects were introduced in the left zone (as described above) for half of the examined subjects and in the right zone for the other half (mirror image of Figure 3).

### 2.3. Data Processing and Statistical Analyses

To code the behaviors on the basis of the recorded material, we used BORIS software (http://www.boris.unito.it), which made it possible to define selected behaviors and to assess their duration and frequency. We scored the behaviors the animals engaged in during the entire experimental trial. Consequently, we were able to assign specific scores to the times of separate bouts of behaviors, their frequency, and the total time an animal spent engaging in a given behavior. The following variables were measured: (1) time spent in the transporter (excluding the latency required to leave the transporter); (2) time spent in the central zone; (3) time spent in the unchanged zone of the chamber; (4) time spent in the changed zone of the chamber; (5) frequency of moving between the zones (left/right/transporter) of the chamber; (6) time spent in contact with the tunnels in the unchanged zone of the chamber; (7) frequency of contact with the tunnels in the unchanged zone of the chamber; (8) time spent in contact with the tunnels in the changed zone of the chamber; and (9) frequency of contact with the tunnels in the changed zone of the chamber. The time spent in the experimental chamber was measured in seconds.

To enhance the legibility of the results and tables, the habituation phase has been indicated as H (the mean score from habituation trials 5 through 7 that presents the outcome of the process of habituation to the experimental environment and served as a reference value for further analyses), while the test trials have been indicated as T1, T2, and T3, respectively. Novelty (i.e., the addition of tunnels in zone C) was introduced in the first test trial (T1). Groups selected on the basis of the activity in the hole–board test were named as follows: the high-activity group was known as HB_H and the low-activity group was known as HB_L.

The data were analyzed using a general linear model procedure (GLM), with repeated measurements (H, T1, T2, T3) as within-subject factors, as well as sex and hole–board group assignment as between-subject factors. This was followed by post-hoc *t*-tests with Bonferroni correction for multiple comparisons. Differences were considered significant for *p* ≤ 0.05.

## 3. Results

The first-step analysis was designed to extract the individuals of the high and low levels of activity (i.e., head-dipping) in the Hole–board apparatus. Table 1 shows the descriptive statistics of this measurement. Individuals falling below the 25th percentile and above the 75th percentile were qualified for further tests.

The complete set of descriptive statistics of all the free-exploration test variables analyzed is shown in Table 2.

### 3.1. Time Spent in the Transporter

The analysis showed significant trial by sex interaction (Wilks’ Lambda; F(3,105) = 7.684; *p* ≤ 0.001; eta^2^ = 0.180), trial by group interaction (F(3,105) = 8.875; *p* ≤ 0.001; eta^2^ = 0.202) and a main effect of the trial (Wilks’ Lambda; F(3,105) = 41.646; *p* ≤ 0.001; eta^2^ = 0.543).

A post-hoc analysis of sex interaction showed a significant decrease in the time spent in the transporter in T1 compared to the habituation phase in females (*p* ≤ 0.001, M_H_ = 65.79, SD_H_ = 22.87, M_T1_ = 29.47, SD_T1_ = 20.30, Cohen’s d = 1.120) and in males (*p* ≤ 0.001, M_H_ = 64.60, SD_H_ = 25.77, M_T1_ = 24.79, SD_T1_ = 10.88′ Cohen’s d = 1.275). In females during the next trials, the amount of time spent in the transporter remained at the same low level. In males, there was an increase in time spent in the transporter between T1 and T3 (*p* ≤ 0.001, M_T3_ = 51.66, SD_T3_ = 21.61, Cohen’s d = 0.861).

A post-hoc analysis of group interaction showed a significant decrease in the time spent in the transporter in T1 compared to the habituation phase in the HB_high group (*p* ≤ 0.001, M_H_ = 55.72, SD_H_ = 17.60, M_T1_ = 23.30, SD_T1_ = 12.64, Cohen’s d = 0.998) and in the HB_low group (*p* ≤ 0.001, M_H_ = 74.67, SD_H_ = 26.36, M_T1_ = 30.96, SD_T1_ = 17.77, Cohen’s d = 1.400).

### 3.2. Time Spent in the Central Zone of the Chamber

The analysis showed a significant main effect of the trial (Wilks’ Lambda; F(3,108) = 20.910; *p* ≤ 0.001; eta^2^ = 0.367), and a main effect of the group (Wilks’ Lambda; F(1,36) = 37.157; *p* ≤ 0.001; eta^2^ = 0.508).

A post-hoc analysis showed a significant decrease in time spent in the central zone in T1 compared to that in the habituation phase (*p* ≤ 0.001, Cohen’s d = 1.102). Subjects from the HB_high group spent less time in that zone than did subjects from the HB_low group (*p* ≤ 0.001, Cohen’s d = 0.964).

### 3.3. Time Spent in the Unchanged Zone of the Chamber

The analysis showed significant sex by group interaction (Wilks’ Lambda; F(3,108) = 4.891; *p* = 0.003; eta^2^ = 0.120), trial by group interaction (F(3,108) = 4.60; *p* = 0.004; eta^2^ = 0.114), trial by sex interaction (F(3,108) = 9.183; *p* ≤ 0.001; eta^2^ = 0.203), and a main effect of the trial (Wilks’ Lambda; F(3,108) = 27.449; *p* ≤ 0.001; eta^2^ = 0.433).

A post-hoc analysis showed a significant decrease in the time spent in the unchanged zone in trial T1 compared to that spent during the habituation in females from the HB_high group (*p* = 0.003, M_H_ = 118.48, SD_H_ = 15.68, M_T1_ = 69.95, SD_T1_ = 29.52, Cohen’s d = 0.696), in males from the HB_high group (*p* ≤ 0.001, M_H_ = 142.64, SD_H_ = 28.92, M_T1_ = 77.35, SD_T1_ = 20.07, Cohen’s d = 0.936), and in males from the HB_low group (*p* ≤ 0.001, M_H_ = 128.81, SD_H_ = 36.47, M_T1_ = 77.28, SD_T1_ = 36.89, Cohen’s d = 0.739), but not in females from the HB_low group. However, there was an increase in time spent in the unchanged zone between T1 and T2 (*p* = 0.008, M_T1_ = 52.20, SD_T1_ = 22.47, M_T2_ = 97.82, SD_T2_ = 36.72, Cohen’s d = 0.654) and between T1 and T3 in that group (*p* ≤ 0.001, M_T3_ = 107.42, SD_T3_ = 38.00, Cohen’s d = 0.792) in females from the HB_low group.

### 3.4. Time Spent in the Changed Zone of the Chamber

Mauchly’s test indicated that the assumption of sphericity had been violated (χ2(5) = 12.43, *p* = 0.029), so the degrees of freedom were corrected using Greenhouse–Geisser estimates of sphericity (ε = 0.85). The analysis showed significant trial by sex by group interaction (F(2.547,108) = 2.893; *p* = 0.048; eta^2^ = 0.074) and a main effect of the trial (F(2.547,108) = 95.496; *p* ≤ 0.001; eta^2^ = 0.726).

A post-hoc analysis showed a significant increase in the time spent in the changed zone in trial T1 compared to the habituation in females from the HB_high group (*p* ≤ 0.001, M_H_ = 158.25, SD_H_ = 28.27, M_T1_ = 279.09, SD_T1_ = 43.54, Cohen’s d = 1.176) and from the HB_low group (*p* ≤ 0.001, M_H_ = 127.66, SD_H_ = 22.00, M_T1_ = 244.77, SD_T1_ = 43.71, Cohen’s d = 1.139), in males from the HB_high group (*p* ≤ 0.001, M_H_ = 113.63, SD_H_ = 22.63, M_T1_ = 254.28, SD_T1_ = 37.00, Cohen’s d = 1.386), and in males from the HB_low group (*p* ≤ 0.001, M_H_ = 85.13, SD_H_ = 17.59, M_T1_ = 223.81, SD_T1_ = 33.10, Cohen’s d = 1.349). There was a decrease in time spent in the changed zone between T1 and T3 in females from the HB_high group (*p* = 0.045, M_T3_ = 219.43, SD_T3_ = 46.72, Cohen’s d = 0.580) and in males from the HB_high group (*p* = 0.035, M_T3_ = 193.41, SD_T3_ = 28.94, Cohen’s d = 0.592). There were also differences between females from the HB_low and the HB_high group in T2 (*p* ≤ 0.001, M_HB_low_ = 186.35, SD_HB_low_ = 33.69, M_HB_high_ = 268.75, SD_HB_high_ = 26.18, Cohen’s d = 0.765).

### 3.5. Frequency of Moving between the Zones (Left/Right/Transporter) of the Chamber

The analysis showed a significant main effect of the trial (Wilks’ Lambda; F(3,108) = 8.930; *p* ≤ 0.001; eta^2^ = 0.199).

A post-hoc analysis showed a significant decrease in frequency of moving between the zones in trial T1 as compared to that during the habituation phase (*p* ≤ 0.001, Cohen’s d = 0.670).

The analysis of between subject effects revealed an interaction effect of sex and group (F(1,36) = 4.146; *p* = 0.049; eta^2^ = 0.103). However, post-hoc comparisons did not reveal any specific differences between the groups.

### 3.6. Time Spent in Contact with the Tunnels in the Unchanged Zone of the Chamber

The analysis showed significant trial by sex by group interaction (Wilks’ Lambda; F(3,108) = 4.193; *p* = 0.008; eta^2^ = 0.104), trial by group interaction (F(3,108) = 4.210; *p* = 0.007; eta^2^ = 0.105), trial by sex interaction (F(3,108) = 9.013; *p* ≤ 0.001; eta^2^ = 0.200), and a main effect of the trial (Wilks’ Lambda; F(3,108) = 15.663; *p* ≤ 0.001; eta^2^ = 0.303).

A post-hoc analysis showed a significant decrease the time spent in contact with the tunnels in the unchanged zone in trial T1 compared to the habituation trials in males from the HB_high group (*p* ≤ 0.001, M_H_ = 98.23, SD_H_ = 23.20, M_T1_ = 51.70, SD_T1_ = 14.57, Cohen’s d = 0.775), but not in males from the HB_low group or in females from either experimental group. An increase in females from the HB-low group in trial T2 (*p* ≤ 0.001, M_T1_ = 30.00, SD_T1_ = 14.16, M_T2_ = 74.88, SD_T2_ = 37.18, Cohen’s d = 0.428) and T3 (*p* ≤ 0.001, M_T3_ = 80.03, SD_T3_ = 32.70, Cohen’s d = 0.833) compared to trial T1 was also shown.

### 3.7. Frequency of Contact with the Tunnels in the Unchanged Zone of the Chamber

The analysis showed a significant main effect of the trial (Wilks’ Lambda; F(3,108) = 15.463; *p* ≤ 0.001; eta^2^ = 0.300), a main effect of sex (F(1,36) = 14.877; *p* ≤ 0.001; eta^2^ = 0.292) and a main effect of the group (Wilks’ Lambda; F(1,36) = 4.724; *p* = 0.036; eta^2^ = 0.116).

A post-hoc analysis showed a significant decrease in frequency of contact with the tunnels in the unchanged zone in trial T1 compared to that in the habituation phase (*p* ≤ 0.001, Cohen’s d = 0.908). Additionally, males more frequently interacted with the tunnels in the unchanged zone than did females (*p* ≤ 0.001, Cohen’s d = 0.610), and subjects from the HB_high group more frequently interacted with the tunnels than did subjects from the HB_low group (*p* = 0.036, Cohen’s d = 0.344).

### 3.8. Time Spent in Contact with the Tunnels in the Changed Zone of the Chamber

Mauchly’s test indicated that the assumption of sphericity had been violated (χ2(5) = 12.085, *p* = 0.034), so the degrees of freedom were corrected using Greenhouse–Geisser estimates of sphericity (ε = 0.85). The analysis showed a significant main effect of the trial (F(2.554,108) = 111.948; *p* ≤ 0.001; eta^2^ = 0.757).

A post-hoc analysis showed a significant increase in time spent in contact with tunnels in the change zone between H and T1 trials (*p* ≤ 0.001, Cohen’s d = 2.664) and between T1 and T3 trials (*p* ≤ 0.001, Cohen’s d = 0.807).

The analysis of between subject effects revealed an interaction effect of sex and group (F(1,36) = 5.164; *p* = 0.029; eta^2^ = 0.125). On the basis of the post-hoc comparisons, it was found that females from the HB_high group spent more time on interaction with the tunnels than did females from the HB_high group (*p* ≤ 0.001, Cohen’s d = 0.882), and males from the HB_high (*p* = 0.002, Cohen’s d = 0.637) and the HB_low (*p* ≤ 0.001, Cohen’s d = 1.011) groups.

### 3.9. Frequency of Contact with the Tunnels in the Changed Zone of the Chamber

The analysis showed significant trial by sex interaction (Wilks’ Lambda; F(3,108) = 5.386; *p* = 0.002; eta^2^ = 0.130), and a main effect of the trial (Wilks’ Lambda; F(3,108) = 6.838; *p* ≤ 0.001; eta^2^ = 0.160).

A post-hoc analysis showed a significant decrease in frequency of contact with the tunnels in the changed zone in females between the T1 and T2 trials (*p* = 0.022, M_T1_ = 9.25, SD_T1_ = 2.42, M_T2_ = 7.25, SD_T2_ = 1.21, Cohen’s d = 0.547).

### 3.10. Effect Size Analysis

To allow the reader to compare the powers of the effects found in our study, Table 3 shows effect size estimations.

A descriptive summary of the results is shown in Table 4.

## 4. Discussion

In this study, we focused on rats’ activity on the hole–board, which we considered to be a type of exploratory activity. This approach is based on a long-standing theoretical tradition [20], which is still being developed nowadays [21]. The general hypothesis was based on the claim that motivational mechanisms are similar in both the response to novelty in a highly familiarized environment and the activity in the hole–board apparatus. If this is true, there should be a strong positive correlation between both of these kinds of activity (namely the response to novelty in the free-exploration box and the activity in the hole–board apparatus). Although the validity of the hole–board protocol for novelty-seeking measurement is often assumed implicitly [22], sufficiently robust evidence to support the above claim is still lacking.

In our study, the scores obtained in the hole–board test allowed us to predict the level of rats’ activity in the free-exploration box only to a very limited extent. The main factor explaining exploratory responses in the free-exploration box was the environmental change that occurred over the course of the experiment. The factors of sex and HB group designation (which indicated high vs. low hole–board activity scores) were of lower predictive value. The direction of the relation between hole–board activity and exploratory scores in the free-exploration box is similar (e.g., individuals that scored high on the hole–board test manifested a high level of exploratory responses, such as time spent in contact with the tunnels). Moreover, HB-high individuals demonstrated a stronger tendency to spend more time in the modified zone of the experimental chamber. As observed by Žampachová et al. [23], behavioral characteristics measured in an open field and the hole–board test share several common properties. An important characteristic of that study was a repeated measure scheme adopted by the authors. The authors drew conclusions about animal personality rather than any specific mechanisms of exploratory behavior. Moreover, their arguments overlapped with exploration understood as a mediator of the adaptation process derived from the theoretical framework of behavioral ecology. However, it has little to do with the theoretical speculation about the mechanism of behavior regulation at an individual level. A similar approach was adopted earlier when researchers tested the temperament concept in rats based on the hole–board procedure. J. Ray and S. Hansen [24] tested rats in the hole–board test and the canopy test six times in a 3-week period. They found the hole–board test to be relevant for assessing the rats’ temperament; the dimension responsible for exploratory behavior, however, was found to be of secondary importance, after harm avoidance. In a further study [25], they found that the relative role of the two aforementioned dimensions changes with ontogenesis. It seems that the prevalent nature of the dimension directly linked to animals’ emotionality, compared to the exploratory (or stimulus seeking) axis, is strictly related to the procedural details, namely the way the animal is placed in the apparatus. The standard procedure involves a human placing the animal in the apparatus’s central zone using their hands. This, in turn, may be recognized as a crucial element of the animal’s situation, meeting the conditions for what is called a “forced exploration” paradigm. As Márquez, Nadal, and Armario [26] have shown, the hole–board procedure involves some level of stress response in tested animals, especially during the first minutes of measurement. This, in turn, would support the view that the hole–board procedure allows researchers to analyze behavioral measures relevant for “active coping” or reactivity rather than exploration per se. To avoid this obstacle, we decided to use a free-exploration procedural variant, which involved a transportation container which was comfortable for the animal being carried, allowing the animal to stay inside or leave at any moment of the trial into the open area of the hole–board. This was intended to address the main legitimate objection voiced by Hughes [4] and Brown and Nemes [5], who suggested that animal activity may be seen rather as an expression of the tendency to escape/leave the apparatus than to explore it. Indeed, our data do not support the view that the exploratory component of behavior repertoire in the hole–board is predominant. On the contrary, the data seem to support the view expressed by Hughes [4] and Brown and Nemes [5]. The reason for this lies in the ecological validity of the two tests: the standard hole–board apparatus vs. the free-exploration chamber [15]. First, the standard hole–board procedure involves testing under daylight conditions, while our free-exploration box is mainly used in darkness. Studying rats under dark conditions is more ecologically valid, as rat are nocturnal animals and typically avoid brightly light places. Secondly, the hole–board procedure is very rarely combined with several habituation trials, which seem unnecessary and unjustified in the light of the simplicity of the environment that the hole–board offers to the animal. However, novelty results from the discrepancy between the previous experience and actual sensory input. The prolonged habituation allows for control of the selective effect of modified parts of the environment. On the contrary, placing the animal in a completely novel test arena does not allow for the attribution of the behavioral activity manifested by an individual tested to a particular stimulation source. Therefore, it may be easily put forward that novel stimulation’s intensity drives an individual to leave the area rather than to explore it. Thirdly, the general structure of the hole–board environment does not offer many affordances. Rather, it offers just one affordance, albeit multiplied.

There is no doubt, however, that the hole–board procedure does measure behavioral responses to a stimulus-rich environment, that it is widely used, and that its reliability is considered sufficient [27]. Nevertheless, one should be cautious when suggesting a theoretical interpretative tool for measuring an animal’s activity in the hole–board apparatus. Based on our experience in studying the exploratory behavior of rats in the free-exploration box [15], we conclude that the hole–board apparatus is not an appropriate tool for measuring exploratory behavior in laboratory rodents. However, we believe that the hole–board procedure may be an attractive tool for behavior analysis in many other fields of study (e.g., [28,29]). What is more, additional data obtained from studies with various variants of hole–board and test conditions (e.g., [30]) are needed to propose more conclusive statements. Nonetheless, this study provides cues for the rethinking of the role of the hole–board procedure as a tool for exploratory activity measurements.

## 5. Conclusions

Based on the results of our study and observations from previous experiments on exploratory behavior in low-stress environments, we must conclude that the hole–board apparatus is not an appropriate tool for measuring exploratory behavior in laboratory rodents. Other behavior regulation mechanisms (e.g., risk assessment, emotional reactivity, active coping) might play a greater role in shaping an animal’s activity in the hole–board apparatus. Our results stress the need for cautious reflection on behavioral tests’ ecological validity when it comes to the studies on animal behavior.

## Figures and Tables

**Table 1:** Descriptive statistics of the rats’ head-dipping activity in the hole–board apparatus (Phase I).

Descriptive Statistics	Female	Male
Valid cases	40	40
Missing cases	0	0
Mean	63.125	65.850
Std. Deviation	12.081	21.832
Minimum	36.000	22.000
Maximum	82.000	117.000
25th percentile	55.750	50.500
50th percentile	66.000	65.000
75th percentile	72.000	80.250

**Table 2:** Descriptive statistics of the variables analyzed in the free-exploration test (Phase II).

Trial	Female				Male			
	HB_H		HB_L		HB_H		HB_L	
	Mean	Stdv	Mean	Stdv	Mean	Stdv	Mean	Stdv
Time spent in the transporter (seconds)								
H	53.489	16.865	78.091	21.987	57.950	18.938	71.255	30.743
T1	25.497	14.998	33.446	24.695	21.100	10.088	28.471	10.855
T2	27.500	11.119	39.975	25.770	38.097	13.819	38.350	25.722
T3	31.250	9.284	19.147	8.359	62.600	20.514	40.725	17.311
Time spent in the central zone of the chamber (seconds)								
H	87.382	17.671	129.922	18.244	103.405	28.115	131.752	31.083
T1	42.113	16.224	86.806	26.956	65.422	28.689	88.218	30.795
T2	44.400	22.570	92.755	31.475	62.176	17.438	90.301	35.716
T3	86.138	40.483	86.452	32.073	83.218	26.809	100.955	30.661
Time spent in the unchanged zone of the chamber (seconds)								
H	118.484	15.684	80.812	18.546	142.643	28.925	128.811	36.466
T1	69.950	29.519	52.200	22.475	77.350	20.072	77.278	36.888
T2	79.050	26.858	97.819	36.724	93.001	31.368	63.225	35.026
T3	80.276	27.460	107.424	38.002	78.147	29.488	81.350	32.115
Time spent in the changed zone of the chamber (seconds)								
H	158.247	28.266	127.658	21.996	113.634	22.628	85.126	17.594
T1	279.091	43.538	244.774	43.715	254.278	37.004	223.808	33.103
T2	268.749	26.178	186.350	33.690	223.852	40.256	226.049	63.430
T3	219.426	46.717	203.725	44.906	193.411	28.945	194.200	48.507
Frequency of moving between the zones (left/right/transporter) of the chamber								
H	16.600	4.537	18.200	2.654	19.100	2.648	18.500	4.238
T1	13.500	5.462	17.000	3.367	15.200	3.011	15.700	2.869
T2	11.800	4.341	16.200	3.490	17.000	3.266	15.400	2.836
T3	15.400	3.596	15.400	2.989	17.000	3.528	15.500	1.780
Time spent in contact with the tunnels in the unchanged zone of the chamber (seconds)								
H	81.598	12.940	47.508	14.835	98.234	23.204	80.869	39.429
T1	48.350	23.602	30.000	14.163	51.704	14.573	48.825	29.438
T2	61.747	22.725	74.880	37.181	67.179	25.447	39.950	32.483
T3	63.954	25.023	80.030	32.701	56.068	20.425	55.650	30.935
Frequency of contact with the tunnels in the unchanged zone of the chamber								
H	6.533	1.492	5.133	0.706	8.733	2.372	7.367	1.222
T1	4.500	1.958	4.700	1.636	6.300	1.567	5.400	1.776
T2	4.800	1.398	4.900	1.370	6.800	1.989	4.600	1.174
T3	4.700	1.337	5.200	0.632	6.100	2.079	5.400	1.174
Time spent in contact with the tunnels in the changed zone of the chamber (seconds)								
H	111.982	19.581	76.694	14.877	75.809	18.746	51.297	17.797
T1	248.350	43.400	200.213	40.112	220.723	40.276	175.604	35.720
T2	243.399	30.898	152.952	32.536	192.424	37.455	182.876	56.932
T3	195.926	46.201	166.971	44.976	164.299	23.887	157.450	48.469
Frequency of contact with the tunnels in the changed zone of the chamber								
H	7.700	1.836	7.500	1.672	7.100	1.277	6.333	1.812
T1	8.400	2.716	10.100	1.853	8.200	2.150	8.900	2.424
T2	6.900	1.370	7.600	0.966	9.600	2.171	8.900	2.726
T3	8.000	2.261	8.500	2.550	9.000	2.867	8.800	3.458

HB_H—high-activity group; HB_L—low-activity group; Stdv—standard deviation; H—habituation trials; T1—first test trial; T2—second test trial; T3—third test trial.

**Table 3:** The ranking list of statistically significant effects based on the partial Eta^2^ values.

Variable	Effect of:	Eta^2^
Time spent in contact with the tunnels in the changed zone of the chamber	trial	0.757
Time spent in the changed zone of the chamber	trial	0.726
Time spent in the unchanged zone of the chamber	trial	0.433
Time spent in the central zone of the chamber	trial	0.367
Time spent in contact with the tunnels in the unchanged zone of the chamber	trial	0.303
Frequency of contact with the tunnels in the unchanged zone of the chamber	trial	0.300
Frequency of contact with the tunnels in the unchanged zone of the chamber	sex	0.292
Time spent in the unchanged zone of the chamber	trial by sex	0.203
Time spent in contact with the tunnels in the unchanged zone of the chamber	trial by sex	0.200
Frequency of moving between the zones (left/right/transporter) of the chamber	trial	0.199
Time spent in the transporter	trial by sex	0.180
Frequency of contact with the tunnels in the changed zone of the chamber	trial by sex	0.130
Time spent in contact with the tunnels in the changed zone of the chamber	sex by group	0.125
Time spent in the unchanged zone of the chamber	trial by sex by group	0.120
Frequency of contact with the tunnels in the unchanged zone of the chamber	group	0.116
Time spent in the unchanged zone of the chamber	trial by group	0.114
Time spent in contact with the tunnels in the unchanged zone of the chamber	trial by group	0.105
Time spent on contact with the tunnels in the unchanged zone of the chamber	sex by group	0.104
Time spent in the changed zone of the chamber	trial by sex by group	0.074

**Table 4:** Descriptive non-statistical summary of the results.

Effect Code	Description of Effect
Time spent in the transporter	
Trial × Sex	The general pattern of the response was similar in both females and males. All subjects spent less time in the transporter in T1. Males, however, spent more time staying in the transporter in T3.
Trial × Group	The general pattern of the response was similar in both HB groups. All subjects spent more time in the transporter in sessions T1 than T3. However, in subjects from the HB-low groups, this tendency was more pronounced.
Time spent in the central zone of the chamber	
Trial	All rats spent less time in the central zone in trial T1 compared to that spent in the habituation phase.
Group	Subjects from the HB_high group spent less time in that zone than did subjects from the HB_low group across all experimental trials.
Time spent in the unchanged zone of the chamber	
Trial × Sex × Group	HB_high females and all males spent less time in the unchanged zone of the chamber in trial T1. However, no such effect was observed in females from the HB_low group. On the contrary—there was an increase in the amount of time spent in the unchanged zone between T2 and T3.
Time spent in the changed zone of the chamber	
Trial × Sex × Group	For all rats, there was an increase in the duration of staying in the changed zone of the chamber in trial T1. However, both females and males from the HB_high groups spent less time in the changed zone in trial T3 as compared to trial T1. The duration of staying in the chamber’s changed zone in HB_high individuals was generally longer than in HB_low individuals, which was most clearly manifested within the female subsample in trial T2.
Frequency of moving between the zones (left/right/transporter) of the chamber	
Trial	For all rats, there was a decrease in the frequency of moving between the zones in trial T1.
Time spent in contact with the tunnels in the unchanged zone of the chamber	
Trial × Sex × Group	HB_high male rats spent less time in contact with tunnels in the unchanged zone, but HB_low male rats and all females did not show this pattern. Females from the HB_low group spent more time in contact with the tunnels in this zone in trials T2 and T3 as compared to trial T1.
Frequency of contact with the tunnels in the unchanged zone of the chamber	
Trial	All rats interacted with the tunnels in the unchanged zone in trial T1 less frequently than they did in the habituation trials.
Sex	Males interacted with the tunnels in the unchanged zone more frequently than females across all experimental trials.
Group	HB_high subjects interacted with the tunnels in the unchanged zone more frequently than their HB_low counterparts across all experimental trials.
Time spent in contact with the tunnels in the changed zone of the chamber	
Trial	All individuals spent more time interacting with the tunnels in the changed zone in trial T1.
Sex × Group	HB_high females spent more time interacting with the tunnels in the changed zone than all other counterparts.
Frequency of contact with the tunnels in the changed zone of the chamber	
Trial × Sex	All subjects responded to tunnel modification with an increase in the frequency of contact with the tunnels in trial T1. However, there was a decrease in the time spent by females on this activity in trial T2.

## Data Availability

All the data are provided in the text.
